# Clinical value of ALU concentration and integrity index for the early diagnosis of ovarian cancer: A retrospective cohort trial

**DOI:** 10.1371/journal.pone.0191756

**Published:** 2018-02-05

**Authors:** Rong Zhang, Wangyang PU, Shuyun Zhang, Li Chen, Weipei Zhu, Li Xiao, Chungen Xing, Kai Li

**Affiliations:** 1 Department of Obstetrics and Gynecology, the Second Affiliated Hospital of Soochow University, Suzhou, China; 2 Department of Oncology, the Second Affiliated Hospital of Soochow University, Suzhou, China; 3 Department of Molecular Medicine Center, the Second Affiliated Hospital of Soochow University, Suzhou, China; 4 Department of General Surgery, the Second Affiliated Hospital of Soochow University, Suzhou, China; Harvard Medical School, UNITED STATES

## Abstract

CA125 amounts have a large overlap in ovarian cancer and benign diseases. We conducted a retrospective cohort trial to assess the clinical value of circulating cell-free DNA concentration and integrity index for the diagnosis of ovarian cancer. A total of 150 patients were recruited. Plasma samples of 24 ovarian cancer patients, 12 benign ovarian cysts, and 12 healthy controls were assessed. By amplifying short ALU-115 repeat and long ALU-219 fragments, circulating cell-free DNA concentrations and integrity index were measured. Plasma ALU-219 fragment levels and integrity index were significantly higher in the ovarian cancer group compared with the benign disease and healthy control groups (p = 0.023 and p = 0.004, respectively). These findings indicated that plasma ALU-219 levels and integrity may have a clinical value in the early diagnosis of ovarian cancer.

## Introduction

Ovarian cancer is one of the most common gynecologic cancers, with high mortality. The cut-off of the conventional ovarian tumor marker CA125 is 35 U/mL, but CA125 would rise in ovarian cancer, endometriosis, and pelvic inflammatory [1]. Since CA125 amounts show a large overlap in different clinical pathological situations, improving the detection rate of ovarian cancer is crucial for timely discovery and prognosis. The identification of the same mutations in ccfDNA and tumor tissue has greatly accelerated the applications of ccfDNA in diagnosis, clinical prevention, and treatment of cancers [2–4]. Moreover, circulating DNA is an effective non-invasive approach to repeatedly determine alterations of tumor genes during the period of patient follow-up [5–7]. In recent years, multiple studies have reported changes of plasma ALU sequence levels and integrity index in a variety of solid tumors, such as colon, thyroid, and breast cancers, suggesting its potential diagnostic value in these malignancies [8–10]. The present study aimed to address the quantitative detection of plasma ALU amounts and integrity index for ovarian cancer diagnosis [11].

Circulating cell-free DNA (ccfDNA) is generally detected by real-time fluorescence quantitative PCR (RT-qPCR), which includes a fluorophore to conventional PCR reactions for real-time monitoring, and absolutely quantifies unknown templates by establishing standard curves. Previous studies suggested the length of ccfDNA fragments released in normal apoptosis is mostly less than 185 bp. Cell in solid tumors after necrosis undergo random digestion by DNA enzymes, and ccfDNA is released into peripheral blood in this process. Indeed, peripheral ccfDNA levels are significantly increased in patients with tumors, showing sequence lengths between 200–400 bp [12]. Therefore, the absolute amounts of long DNA fragments and relative ratio of long to short fragments, as well as the integrity index, may reflect their possible origin, cancerous or not. Based on previous studies [13], we used the RT-qPCR method, amplified ALU-115 and ALU-219 sequences to detect plasma DNA concentrations and integrity index in ovarian cancer samples, comparing ALU with the conventional tumor marker CA125 in ovarian cancer diagnosis. Our data showed that serum ALU-219 concentrations and the integrity index of ALU-219/ALU-115 may be used to differentiate patients with ovarian cancer from those with ovarian benign cyst and healthy controls.

## Materials and methods

This was a retrospective cohort trial, with three parallel groups. The protocol was provided as S1 File and S2 File. The study was approved by the national committee for clinical drugs trials of the second affiliated hospital of Soochow University. The included patients were informed and consented with the grouping according to the "Declaration of Helsinki". The trial was registered and the number was ChiCTR-DDT-12002966 (http://www.clinicaltrialecrf.org). All participants provided written informed consent before enrollment.

### Study population

**Fig 1:**
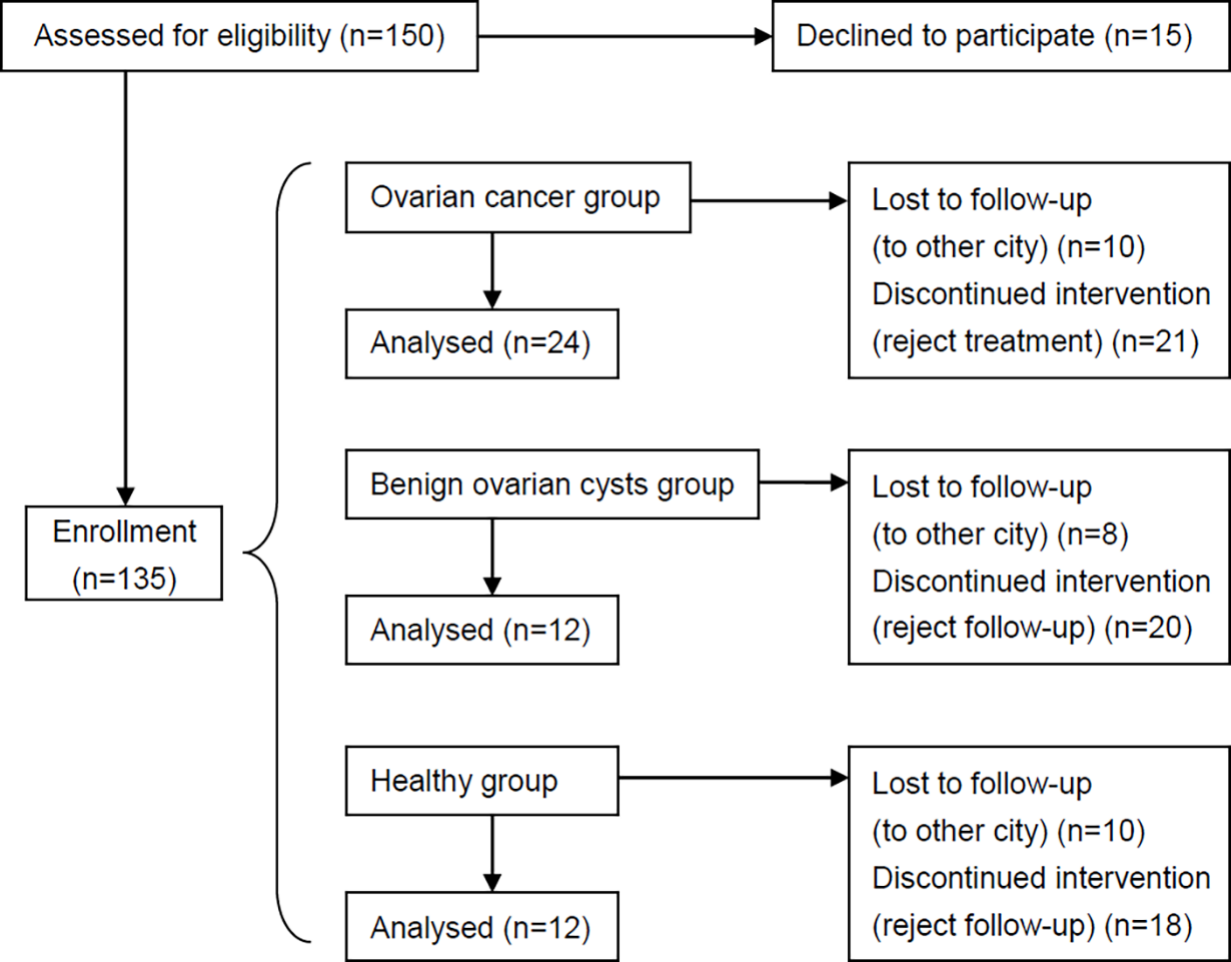
CONSORT flow diagram of patient participation and follow up.

This was a retrospective cohort trial. A total of 150 participants (Fig 1) including ovarian cancer patients, benign ovarian cyst patients and healthy controls were recruited in the second affiliated hospital of Soochow university, between December 2012 and October 2013. Ovarian cancer patients were tested pre-operatively by CA125 amounts and imaging, and pathologically confirmed post-operatively by FIGO I-IV staging. This group received standard cytoreductive surgery for ovarian cancer and postoperative 6 chemotherapy cycles of 21 days. The benign ovarian cyst group was ruled out for possible malignancy clinically and eventually pathologically confirmed after surgery. This group was treated with oophorocystectomy or single accessory resection, and followed up for 6 months. Healthy controls had undergone routine physical examination before inclusion, and were ruled out for ovarian disease and other cancers, and followed up for 6 months. Due to reasons (not meeting inclusion criteria, loss to follow-up and no local medical insurance reimbursement), complete preoperative and postoperative blood samples were obtained from 24 ovarian cancer (ovarian serous adenocarcinoma, 21 cases; clear cell carcinoma, 2 cases; endodermal sinus tumor, 1 case), 12 benign ovarian cyst (ovarian chocolate cyst, 5 cases; mucinous cyst adenoma, 3 cases; mature teratoma, 2 cases; serous cystadenoma, 1 case; fibroma, 1 case), and 12 healthy control patients. Patient ages in the three groups were similar.

### Reagents and instruments

DNA extraction kit (QIAamp Circulating Nucleic Acid Kit) and SYBR Green PCR mix were purchased from QIAGEN (QIAGEN Group, Germany). Primers were synthesized by Shanghai Sangon Biotech Company (China). The Bio-Rad CFX96 Real-time PCR System fluorescence quantitative PCR instrument was purchased from Bio-Rad Laboratories Co., Ltd. in USA.

### Plasma preparation for ALU-qPCR

A total of 6 ml of peripheral blood was collected in an EDTA-containing tube. Samples were transferred to the study laboratory within 4 h for processing. After centrifugation at 3000 r/min for 10 min the plasma was collected, of which 2 ml specimens were stored at -80°C. Then, 2 ml of plasma was handled in accordance with instructions of the QIAamp Circulating Nucleic Acid kit. The extracted DNA was ultimately stored at -80°C after elution with 50 μL elution buffer. Meanwhile, another 2 mL of peripheral blood was collected with EDTA free tubes to assess serum CA125.

### Quantitative PCR of ALU repeats

Two primer pairs were used, respectively, for the amplification of short and long ALU repeats. The results obtained with the ALU-115 primers reflect total ccfDNA amounts, while those of ALU-219 primers represent the amounts of DNA released from tumor cells. The long to short fragment ratio reflected ccfDNA integrity. ALU-115 primers were as follows: forward, 5'-CCTGAGGTCAGGAGTTCGAG-3' and reverse, 5'-CCCGAGTAGCTGGGATTACA-3'; ALU-219 primers were as follows: forward, 5'-CACGCCTGTAATCCCAGCACTTT-3' and reverse, 5'-ATCTCGGCTCACTGCAACCTCC-3'. ALU-PCR amplification system consisted of 12.5 μL QuantiFast SYBR Green PCR mix, 0.5 μL of forward and reverse primers, 1 μL template of ccfDNA, and 10.5 μL RNase-free water in a total 25μL volume. ALU-PCR amplification was performed by initial denaturation at 95°C for 5 min, followed 40 cycles of denaturation at 95°C for 20 s, and annealing and extension at 65°C for 30 s. Two ml samples of peripheral blood from healthy individuals were used for genomic DNA extraction. The absolute amounts of ccfDNA in each sample was determined by a standard curve using 5 serially diluted concentrations from 52 to 0.0052 ng/μL for PCR.

**Fig 2:**
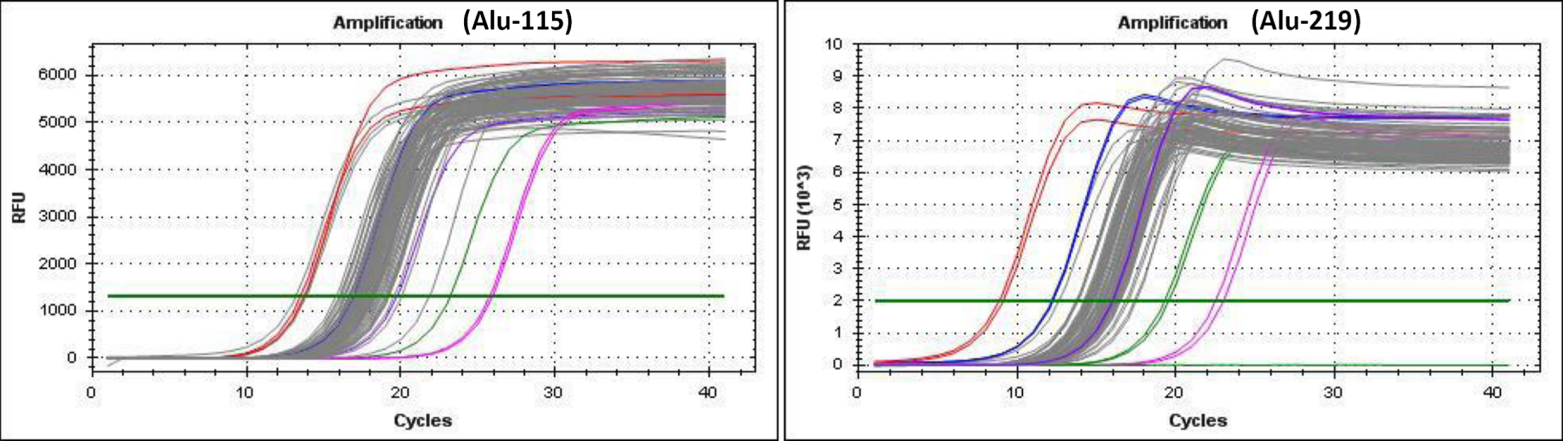
Representative amplification curves for ALU-115 and ALU-219 obtained by RTFQ-PCR. Colored and grey represent standard curves and individual ccfDNA samples, respectively. Amplification efficiency = 94.1%-113.1%, R2 = 0.985–0.999.

A negative control (without the template) was employed on each plate. All PCR assays were performed in a blinded fashion, not knowing the specimen identity. Amplification curves were obtained to confirm the accuracy of amplification signals (Fig 2).

### Statistical methods

Statistical analyses were performed with the SPSS 18 package. As preliminary evaluation showed integrity index and age were normally distributed, we employed an analysis-of-variance to assess possible differences among the three groups. ALU-115, ALU-219, and CA125 showed a skew distribution pattern, and inter-group differences were evaluated by Mann-Whitney U test. Areas under the receiver-operating characteristic curves (AUC-ROC) were calculated to evaluate the diagnostic or predictive performance of ALU-115, ALU-219, and integrity index in ovarian cancer. Spearman correlation analysis was used to assess the associations of serum ALU-115, ALU-219, integrity index, and CA125 with ovarian cancer. Two tailed P<0.05 was considered being statistically significant.

## Results

### Clinical characteristics of patients

**Fig 3:**
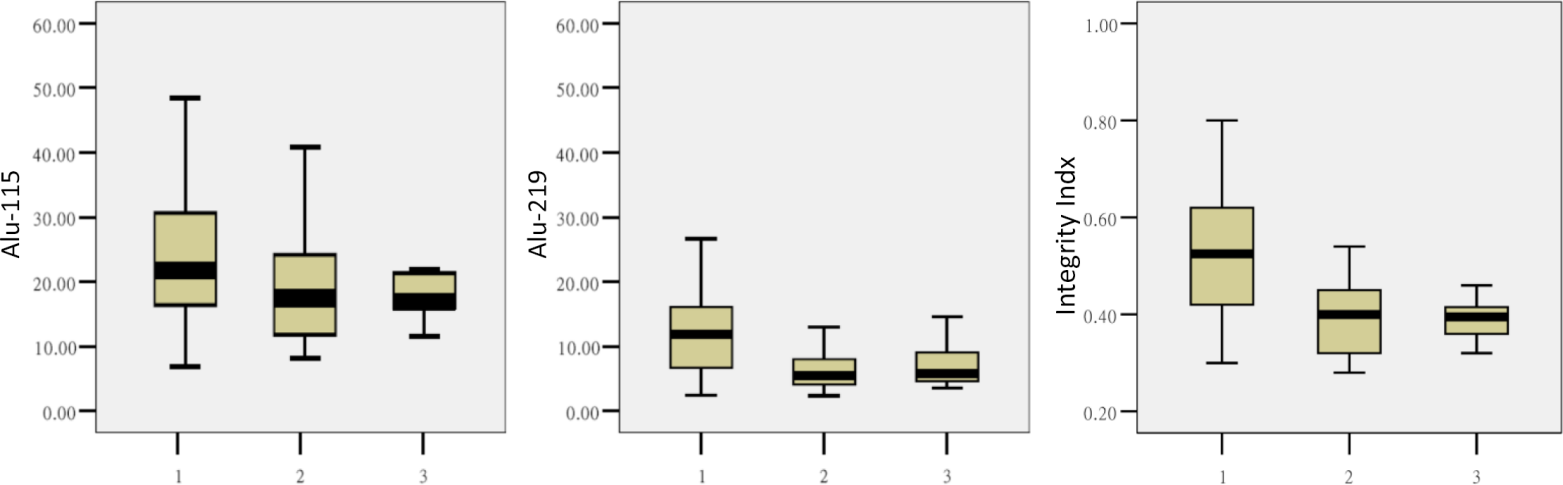
Amounts and integrity of plasma ALU. The numbers 1, 2, and 3 on the X axis refer to ovarian cancer, ovarian benign cyst and healthy control patients, respectively. Concentrations and integrity of plasma ALU in ovarian cancer patients were significantly higher than those of the ovarian benign cyst and healthy control groups.

The general information of patients is shown in Tables 1 and 2. Age and integrity index showed normal distribution, and were presented as mean ± standard deviation (SD). ALU-115/ALU-219 concentrations and CA125 showed skew distribution, and were presented as median (P25-P75). The preliminary evaluation of ALU-115 concentration, ALU-219 concentration, and integrity index is shown in Fig 3.

**Table 1:** General information and inter-group analysis of age and integrity index.

	Age		ANOVA	P	Integrity index		ANOVA	p
	mean±SD	95% CI			mean±SD	95% CI		
			F = 2.886	0.066			F = 6.152	0.004*
**Ovarian cancer** ^**1**^ **n = 24**	51.96±14.12	46.00–57.92	1 ***vs*. 2**	0.054	0.53±0.16	0.47–0.60	1 ***vs*. 2**	0.027*
**Benign cyst** ^**2**^ **n = 12**	42.67±12.28	34.86–50.47	2 ***vs*. 3**	0.976	0.41±0.11	0.34–0.48	2 ***vs*. 3**	0.099
**Normal** ^**3**^ **N = 12**	37.00±13.07	34.96–50.71	1 ***vs*. 3**	0.058	0.40±0.06	0.36–0.43	1 ***vs*. 3**	0.002*

* P <0.05, statistically significant. 1: Ovarian cancer group, 2: Benign cyst group, 3: Normal group.

**Table 2:** General information and inter-group analysis of ALU-115 /ALU-219 /CA-125.

	115(ng/μL)		M-W	P	219(ng/μL)		M-W	P	CA-125		M-W	P
	M(P25-P75)	95% CI	Z		M(P25-P75)	95% CI	Z		M(P25-P75)	95%CI	Z	
**ovarian cancer** ^**1**^ **n = 24**	19.44 (13.74–29.80)	16.71- 37.01	1 *vs*. 2 -1.443	0.149	11.88 (6.46–16.31)	7.27- 35.46	1 *vs*. 2 -2.215	0.027*	474.35 (78.23–1627.75)	12.21- 23.94	1 *vs*. 2 -4.027	<0.0001*
**benign cyst** ^**2**^ **n = 12**	14.92 (8.33–24.41)	8.81- 17.62			5.46 (3.71–8.24)	3.39- 11.74			27.56 (17.65–57.21)	4.93- 9.29		
**Norma**^**3**^ **N = 12**	14.41 (13.33–19.27)	490.29- 1783.90	2 *vs*. 3 -0.520	0.603	5.75 (4.56–9.71)	19.28- 51.71	2 *vs*. 3 -0.462	0.644	18.38 (11.19–25.24)	13.60- 23.90	2 *vs*. 3 -1.848	0.065

Kruskal-Wallis H analysis on ALU-115/ALU-219/CA-125 in the three groups was x^2^ = 2.932, p = 0.231; x^2^ = 7.516, p = 0.023*; x^2^ = 28.98, p = 0.000*, respectively.

*P<0.05, statistically significant.

M-W refers to Mann-Whitney U analysis.

### Inter-group differences of age, ALU-115, ALU-219, integrity index, and CA125

Integrity index was significantly increased in the ovarian cancer group compared than the benign and normal groups (Table 1) as analyzed by ANOVA. The retrospective power analysis on integrity index is 0.937 (Type I error = 0.05). ALU-219 and CA125 were significantly different by Kruskal-Wallis H test (Table 2), while ALU-115 showed no significant difference among the three groups. Further inter-group differences were assessed by Mann-Whitney U test, and significant differences were found in ALU-219 and CA125 levels between ovarian cancer patients and the benign ovarian cyst group. No significant differences in ALU-115, ALU-219, and CA125 were obtained between the benign ovarian cyst and normal groups. ALU-219 and CA125 medians in ovarian cancer patients were higher compared with the benign and normal groups, with significant differences.

### Associations of plasma ALU-115, ALU-219, and integrity index with CA125 in ovarian cancer patients

As evaluated by Spearman correlation analysis, no associations ALU-115, ALU-219, and integrity index with CA125 were observed (r = -0.0086, P = 0.689; r = 0.027, P = 0.899; r = 0.207, P = 0.333; respectively).

### Diagnostic values of ALU-115, ALU-219, and integrity index in ovarian cancer

**Fig 4:**
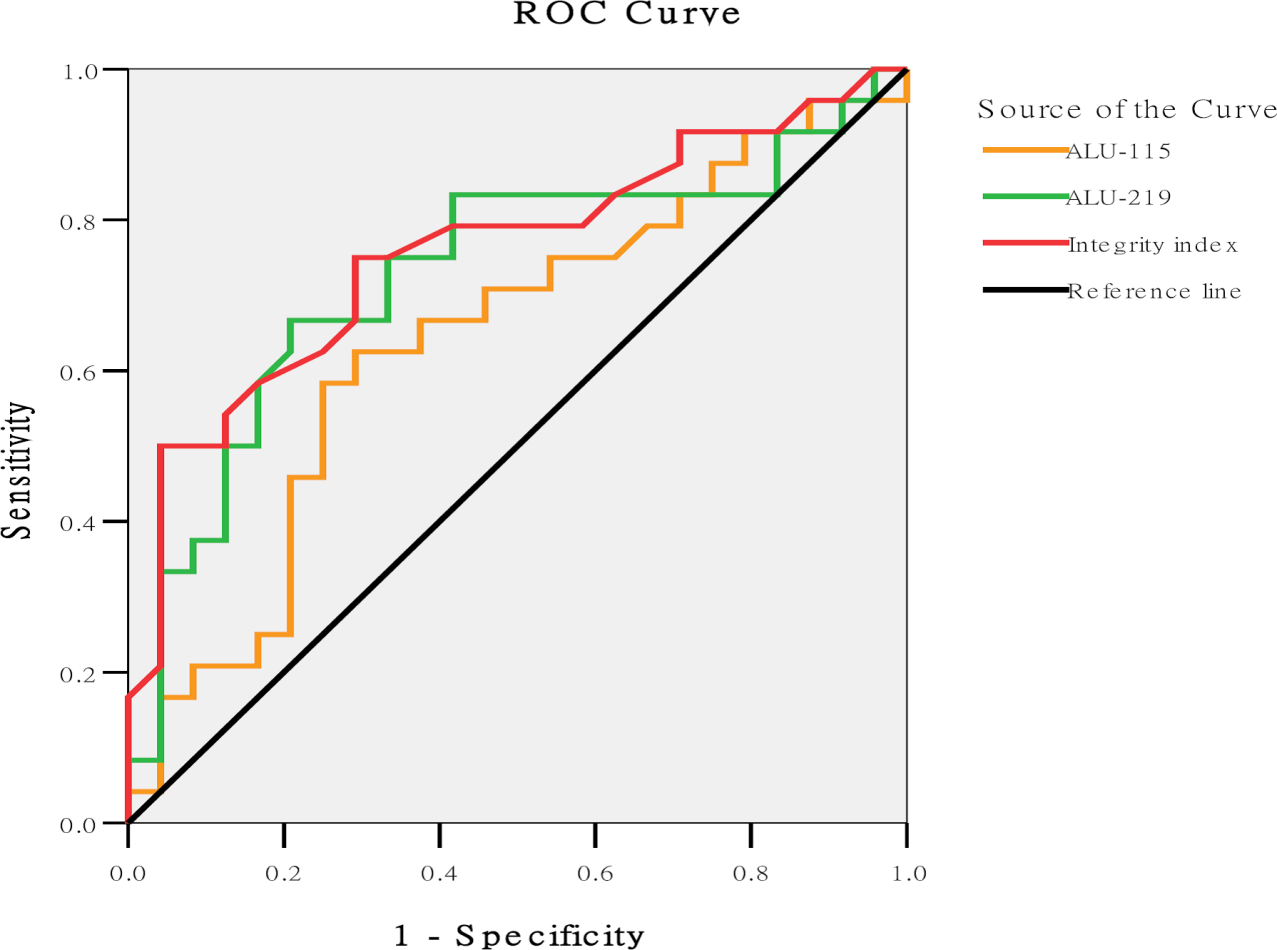
ROC for distinguishing patients with ovarian cancer from healthy subjects using the ALU-115/ALU-219 integrity index.

ROC curve analyses (The healthy controls and benign samples were lumped together for this analysis)were performed to determine the diagnostic values of ALU-115, ALU-219, and integrity index in ovarian cancer (Fig 4). The AUC of ALU-115, ALU-219, and integrity index were 0.641 (95%CI = 0.482–0.801), 0.730 (95CI% = 0.581–0.879), 0.759 (95%CI = 0.619–0.898), respectively, and the P values were 0.093, 0.006, and 0.002, respectively, compared with AUC = 0.5. The AUC of ALU-219 and integrity index combined predicting ovarian cancer is 0.792 (95%CI = 0.654–0.929) compared with AUC = 0.5(P = 0.001) by SPSS analysis.

According maximum Youden’s index, the cut-off values for ALU-115, ALU-219, and integrity index were 17.28 ng/μL, 8.57 ng/μL, and 0.56, respectively (Table 3). Further evaluation showed that the coincidence rate of using cut-off values for ALU-219, integrity index, and CA125 to detect patients with benign ovarian cysts and normal individuals were 0.79 (19/24), 0.96 (23/24), and 0.83 (20/24), respectively. The coincidence rate of integrity index was higher than that of the CA125. It indicated that if integrity index is less than 0.56, the possibility of benign ovarian tumor is higher, suggesting integrity index is expected to be used singlely in the differential diagnosis of benign and malignant ovarian diseases to some extent.

**Table 3:** The best cutoffs of the sensitivity, specificity and Youden index for ALU-115, ALU-219, and integrity index.

	sensitivity	specificity	Youden index
**ALU-115**	0.625	0.708	0.333
**ALU-219**	0.667	0.792	0.459
**integrity index**	0.75	0.708	0.458

## Discussion

In this exploratory research, we sought to preliminarily confirm the potential diagnostic values of ccfDNA circulating in plasma for ovarian neoplasms. The main findings were that serum ALU-219 concentration and the ALU-219/ALU-115 integrity index may be useful to differentiate patients with ovarian cancer from ovarian benign cyst and healthy control subjects.

CcfDNA remains at low and constant levels in healthy individuals, but increases significantly in patients with various diseases, including tumors. In this study, ALU-219 levels in the ovarian cancer group were higher than those of the benign ovarian cyst and normal groups (p = 0.023); meanwhile, the benign ovarian cyst and normal groups showed no significant difference (p = 0.644). ALU integrity index in ovarian cancer patients was higher than in the benign ovarian cyst and normal groups (p = 0.004), with no significant difference between the benign ovarian cyst and normal groups (P = 0.099). We did a retrospective power analysis on integrity index according to the current sample size and the test level. The retrospective power analysis on integrity index is 0.937 (Type I error = 0.05). When power is >0.9, the conclusion is more credible. It shows that integrity index has a high power for distinguishing between benign and malignant tumors under the premise of the current sample size and the conclusion is credible.

ALU and CA125 were not significantly correlated as assessed by Spearman correlation analysis, likely because of their different metabolic dynamics patterns. The AUC of ALU-219 and integrity index were greater than 0.73 and P values were less than 0.05, suggesting that single or combined use of ALU-219 and integrity index contributes to the diagnosis of ovarian cancer, a high specificity of integrity index with the potential to become a new ovarian cancer marker independent of existing markers.

Currently, pathological diagnosis is the "gold standard" of the diagnosis of malignancies, including ovarian cancer. However, because of the deep anatomical location of the ovary, many ovarian cancer patients cannot be pathologically confirmed with certitude at the early stage before surgery. Extensive efforts have been made to approach the early diagnosis of ovarian cancer through imaging combined with related tumor markers; however, poor sensitivity and specificity led to relatively high risks of misdiagnosis and mistreatment in certain cases. Local biopsy on one hand is too painful for long-term follow-up monitoring; on the other hand, it is difficult to obtain all the tumor traits with just a small amount of tissue. Therefore, it is clinically required to provide early, effective, and minimally invasive diagnosis tools with high sensitivity and specificity. Nearly 60 years have elapsed since peripheral free DNA was found in 1948 [14]. In the past 20 years, the same genetic trait was discovered for the peripheral ccfDNA as that from the tumor tissue [15]. Therefore, ccfDNA has the potential to replace existing detection methods for the diagnosis and efficacy evaluation of clinical tumors, or as an alternative choice for early diagnosis of cancer.

## Conclusions

Studies showed that blood ccfDNA has significant associations with incidence, improvement, and relapse of some malignancies [16]. Our findings should be validated in a prospective study with a large number of patients receiving more homogeneous experimental methods. In conclusion, this study suggested that circulating ALU-219 concentration and the ALU-219/ALU-115 ratio (ccfDNA integrity index) could be used as diagnostic markers in ovarian cancer. Monitoring ALU level in plasma could be applied for subsidiary diagnosis and prognosis of ovarian cancer, alone or in combination with other tumor markers.

## Supporting information

S1 File Trial study protocol (English edition). Trial study protocol-Eng.doc.
(DOC)

S2 File Trial study protocol (Chinese edition). Trial study protocol-Chs.doc.
(DOC)

S3 File Flow chart. Flow chart.pdf.
(PDF)

S4 File Financial supports. Financial supports.pdf.
(PDF)
