# Supplementary material for: Clinical value of ALU concentration and integrity index for the early diagnosis of ovarian cancer: A retrospective cohort trial
Source: PLoS One. 2018 Feb 5;13(2):e0191756. doi: 10.1371/journal.pone.0191756 (PMC5798773; doi:10.1371/journal.pone.0191756)
Supplement: S1 File — Trial study protocol-Eng.doc. (DOC) [file pone.0191756.s001.doc]

**Trial study protocol**

Application of Peripheral Blood Circulating DNA as Biomarker in Ovarian Cancer

Objective: To evaluate the value of peripheral blood free DNA as a biomarker in ovarian cancer

Test Category: Diagnostic Test

Applicant: Soochow University, China

Research Unit: the Second Affiliated Hospital of Soochow University

Responsible person: Li Kai

Main Researcher: Zhang Rong

Test date: November 11 to November 2017

Contact: Zhang Rong

Tel: 86-0512-67784769

**1. Background**

Ovarian cancer is one of the common malignant tumors of gynecology and its mortality ranks first in gynecological tumors. 70% patients who were diagnosed as ovarian cancer are at late stage (Ⅲ and Ⅳ). Although by the active treatment, 5-year survival rate of ovarian cancer is only 15% to 20%. If the patients can be diagnosed at early stage(Ⅰ) , the 5-year survival rate is up to 77 % ~ 87%. If the patients at well-differentiated stage Ⅰ, 5-year survival rate can reach 94% [1]. Therefore, the key to improving survival is early diagnosis. Specific and sensitive tumor markers play an important role in the diagnosis of ovarian cancer.

In recent years, the detection of free DNA in peripheral blood of patients with cancer has provided a new means for early diagnosis and relapse monitoring of tumor. Free DNA can exist in blood, cerebrospinal fluid and synovial fluid and other body fluids, is a kind of free form of outside DNA of the cells, also known as circulating free DNA or circulating DNA. As early as 1947, Mandel et al [2] found the presence of free DNA in human peripheral blood. Since 1977 Leon et al [3] found that the plasma DNA content of cancer patients was significantly higher than normal, the tumors research with free DNA has become a hot point. **Qualitative studies of free DNA have shown that plasma DNA in cancer patients has a genetic abnormality consistent with tumor cell DNA [4-7].**

Proto-oncogene mutations, tumor suppressor gene methylation changes and microsatellite instability changes [8-9] can predict the prognosis of cancer patients [10]. At present the study of free DNA in blood is focused on the "multiple disease" such as lung cancer, rectal cancer, breast cancer, prostate cancer and melanoma, and there is little research on ovarian cancer. Hagiwara et al [11] explored on DNA encoding 248 and 249 mutation detection in smokers of lung cancer, non-smokers of lung cancer and healthy individuals, and found that DNA p53 gene mutation can be used as a predictor of lung cancer. Ryan et al [12] researched on a large number of colon cancer patients with free DNA. It has shown that free DNA k-ras gene mutation in patients with tumor recurrence rate were up to 62.5%. A prospective cohort study by European cancer and nutrition organization collected the free DNA of quasi-healthy person and cancer occurrence in themselves, including bladder cancer, lung cancer, Upper gastrointestinal cancer, etc. **The study found that before 18. 3 months clinical diagnosis the tumor, free DNA in the blood can be detected in the corresponding gene mutation**(k-ras gene 12 codon and p53 gene mutation detection), and predictability is associated with the primary site, type and stage of the tumor.

Frattini et al. [13] showed that free DNA combined detection of quantification, qualitative and site-directed would improve the clinical diagnosis of the tumor. This study observed changes in free DNA levels, k-ras and p16 levels in free DNA before and after surgery in patients with colorectal cancer. They found that postoperative free DNA levels decreased gradually, and when the tumor relapsed rapidly, the mutant k-ras and p16 gene methylation could again be detected. MelNikov [14]used methylated-specific PCR to detect the methylation differences in tumor tissue and plasma of ovarian cancer patients, found that combined detection BRCA1, HIC1, PAX5, PGR and THBS1 gene, the diagnosis sensitivity of ovarian cancer was 85%, specificity was 61%, and its efficiency was comparable to that of direct application of tumor tissue. Dobrzycka [15] used PCR-RFLP method to evaluate the plasma free DNA and ovarian cancer progression, found that it was related in the increased free DNA and the decreased tumor survival rate. Based on the above findings, we hope that similar studies can be used to find the changes of free DNA levels and related genes in ovarian cancer patients, to obtain the relationship between the free DNA and the pathological changes of ovarian cancer in Chinese population, and to provide reference for ovarian cancer prevention and a genetic diagnostic method for relapse monitoring through quantitative indicators.

The current study shows that the changes in free DNA and ovarian cancer are closely related. The discovery and research of free DNA provides the possibility of noninvasive diagnosis and monitoring of the disease. It can solve the problem that tumor gene detection can only be restricted by tumor tissue, which provides a simple way for the diagnosis, treatment monitoring and prognosis of tumor. However due to the small amount of free DNA, it is more difficult and expensive to extraction of free DNA. Now it exist that free DNA detection methods are different, the sensitivity is difference, and free DNA detection has low specificity. Therefore it is important to establish a practical test method in order to facilitate the clinical use of free DNA.

In this study, the concentration of free DNA in free blood was detected by nested PCR and methylation specific fluorescence PCR. The plasma free DNA concentration in ovarian cancer patients, ovarian benign cysts and healthy women was compared and detected the methylation status of hot genes in order to detect the correlation between peripheral blood DNA and ovarian cancer tissues. Looking to the future, the basis of ovarian cancer free DNA and clinical research will become a hot topic of attention.

**2. Purpose**

To evaluate the significance of plasma free DNA as a biomarker in the diagnosis and prognosis of ovarian cancer.

**3. Test design**

This trial is a retrospective cohort study.

**4. Subject selection and withdrawal**

A. Inclusion criteria：

Gender：female

Age：20-80 years

1）Ovarian cancer group：

Ovarian cancer with pathologic diagnosis.

Sign informed consent.

2）Benign ovarian cyst group：

Benign ovarian cyst group with pathologic diagnosis.

Sign informed consent.

B. Exclusion criteria：

1）Combined with other malignancies.

2）Pregnancy.

3）Combined with autoimmune diseases.

C. Subjects withdrew in the middle of the test.

1）It is necessary to suspend the trial based on the medical caused by researchers.

2）The patient himself asked to stop the trial.

**5. Number of cases and grouping**

All participants were averagely divided into three groups which ovarian cancer group (n=50), benign ovarian cysts group (n=50) and healthy group (n=50).

**6. Clinical trial steps:**

**（A）Collect specimens**

1. Ovarian cancer group

1) Six ml of peripheral blood was collected in an EDTA-containing tube. Samples were transferred to the study laboratory within 4 h for processing.

2) Collect fresh ovarian cancer specimens 1*1*1cm^3^ intra-operation and froze at -80 ℃.

3) Test free DNA concentration and integrity in the first week after operation. The follow-up criteria for ovarian cancer is once a month in the first year, once every three months in the second year, once every six months in the third year, once an annual in the 4-5 years after surgery. Blood samples were collected at the time of follow-up to test free DNA concentration and integrity, blood routine, biochemical routine, CA-125 and clinical imaging or gynecological color Doppler ultrasonography. The whole stage of follow-up is 5 years.

2. Benign ovarian cysts group：

1) Six ml of peripheral blood was collected in an EDTA-containing tube. Samples were transferred to the study laboratory within 4 h for processing.

2) Collect fresh ovarian cancer specimens 1*1*1cm^3^ intra-operation and froze at -80 ℃.

3) The follow-up criteria for benign ovarian cysts is once a month after surgery in six months, then once an annual in 5 years. Free DNA concentration and integrity, CA-125 and imaging or gynecological color Doppler ultrasonography were measured at each time of follow-up.

3. Healthy group

Six ml of peripheral blood was collected in an EDTA-containing tube. Samples were transferred to the study laboratory within 4 h for processing. Free DNA concentration and integrity was measured at once an annual in 5 years.

1. **Test free DNA concentration and integrity**

1. Samples were transferred to the study laboratory within 4 h for processing.

2. The plasma samples were centrifugation at 3000 r/min for 10 min. Three ml of plasma was collected and froze at -80 ℃.

3. Two ml of plasma was handled in accordance with operation instructions of QIAamp Circulating Nucleic Acid kit.

4. Two primer pairs were used respectively for the amplification of the short and long ALU repeats. The results obtained using the ALU-115 primers reflect the total ccfDNA, while the results of ALU-219 primers represent the amount of DNA released from tumor cell. The ratio of longer to shorter fragments used to represent the integrity of ccfDNA. ALU-115 primers were as follows: forward, 5' –CCTGAGGTCAGGAGTTCGAG-3' and reverse, 5' –CCCGAGTAGCTGGGATTACA-3'; ALU-219 primers were as follows: forward, 5' –CACGCCTGTAATCCCAGCACTTT-3' and reverse, 5'-ATCTCGGCTCACTGCAACCTCC-3'. Integrity of ccfDNA =ALU219-qPCR/（ALU115-qPCR+ ALU219-qPCR).

5. Detection of ccfDNA methylation status in blood and ovarian cancer specimens: Nested PCR was performed by using high-fidelity DNA polymerase, and primers were designed according to according to the literature and information in NCBI. The PCR product was sequenced. According to the sequencing results, the primers were designed and the methylated specific quantitative PCR was used to extract the plasma free DNA from healthy people, ovarian benign cysts patients and ovarian cancer patients before and after operation, and evaluate whether there was a significant difference in the number of free DNA methylation between groups.

**（C）Indicators**

1) Plasma free DNA concentration and integrity

2) Ovarian cancer-related gene methylation status.

**7. Statistical Analysis**

Statistical analyses were performed with SPSS 18 package. Possible differences among the three groups were tested by Mann-Whitney U tests. The area under the curve of receiver-operating characteristics (AUC-ROC) was calculated for evaluating the diagnostic or predictive performance of ALU-115, ALU-219, and integrity index in diagnosing ovarian cancers. Spearman correlation analysis was used to test the relationship between serous ALU-115, ALU-219, integrity index, and CA125 of ovarian cancer patients. P <0.05 (two tailed) was considered being statistically significant.

**8. Quality control and assurance**

During the course of this study, the clinical investigators assigned by the applicant will conduct on-site monitoring visits to the study hospitals on a regular basis to ensure that all the contents of the research program are strictly adhered to and filled in the correctness of the research data. Participate in the study staff must undergo a unified training, unified record and strict standard. The entire clinical trial process should be carried out under strict operation. Researchers should fill in the requirements in accordance with the case report form. Truthful, detailed and careful record of the CRF contents ensure that the case reports are true and reliable. All observations and findings in clinical trials should be verified to ensure data reliability and to ensure that the conclusions of the clinical trial are derived from the original data. In the clinical trial and data processing stage have the appropriate data management measures.

**9. Ethical requirements**

This clinical trial must follow the Helsinki Declaration and the Chinese clinical trials research regulations. Before the start of the trial, the approval of the Audit Committee of the Second Affiliated Hospital of Soochow University was obtained. Each patient is enrolled in this study and it is the responsibility of the research physician to provide a complete and comprehensive introduction to the purpose, procedure and possible risks of the study in written form to the person or its designated representative. It should let the patients know that they have the right to withdraw from the study at any time. A written patient's informed consent form (included in the appendix) is required for each patient prior to enrollment. It is the responsibility of the research physician to obtain informed consent before each patient enters the study and retain it with the study file.

**Data saved:** destroyed after use

**References**

[1] Vergot e I, De Brabant e J, Fyles A, et al. Prognostic importance of degree of differentiation and cyst rupture in stage Ⅰ invasive epithelial ovarian carcinoma [J]. Lancet, 2001, 357(9251): 176-82.

[2] Mandel P, Met ais P. Les acides nucleiques du plasma sanguinchez I, home [J]. C R Acad Sci Paris, 1948, 142: 241-43.

[3] Leon SA, Shapiro B, Sklaroff DM, et al. Free DNA in the serum of cancer patient s and the effect of therapy [J]. Cancer Res, 1977, 37: 646- 50.

[4] Mirza S, S harma G, Prasad CP, et al. Promoter hypermethylation of TMS1, BRCA1, Eralpha and PRB inserum and tumor DNA of invasive ductal breast carcinoma patients [J] . Life Sci, 2007, 81(4): 280-7.

[5] Weaver KD, Grossman SA, Herman JG. Methylated tumor specific DNA as a plasma biomarker in patients with glioma [J]. Cancer Invest, 2006, 24(1): 35-40.

[6] Yang H J, Liu V W, Wang Y, et al. Detection of hypermethylated genes in tumor and plasma of cervical cancer patients [J] .Gynecol Oncol,2004, 93(2): 435-40.

[7]Sanchez-Cespedes M, EstellerM, Wu L, et al. Gene promoter hypermethylation in tumors and serum of head and neck cancer patients[J]. Cancer Res, 2000, 60(4): 892-95.

[8] Anker P, Stroun M. Circulating DNA in plasma or serum [J]. Medicina( B A ires), 2000, 60( 5Pt2) : 699-702.

[9] Shapiro B, Chak rabarty M, Cohn EM, et al. Determination of circulating DNA levels in patients with benign or malignant gastro intestinal disease[J] . Cancer, 1983, 51(11): 2116-20.

[10] Sozzi G, Conte D, Mariani L, et al. Analysis of circulating tumor DNA in plasma at diagnosis and during follow-up of lung cancer patients[J] . Cancer Res, 2001, 61(12): 4675-8.

[11] Hagiwara N, Mechanic LE, Trivers GE, et al. Quantitative detection of p53mutat ions in plasma DNA from tobacco smokers[J] . Cancer Res, 2006, 66(16): 8309-17.

[12] Ryan BM, Lefort F, M cmanus R, et al. A prospective study of circulating mutant KRAS2 in the serum of patients with colorectal neoplasia strong prognostic indicator in postoperative follow up [J] .Gut, 2003, 52(1): 101-8.

[13] Frattini M, Gallino G, Signoroni S, et al. Quantitative and qualitative characterization of plasma DNA identifies primary and recurrent colorectal cancer [J]. Cancer Lett, 2008, 263(2): 170-81.

[14]B. Dobrzycka, S. J. Terlikowski, M. Kinalski, et al. Circulating free DNA and p53 antibodies in plasma of patients with ovarian epithelial cancers[J]Ann. Oncol. 2011,22: 1133-40.

[15]A Melnikov, D Scholtens, AGodwin, et al. Differential Methylation Profile of Ovarian Cancer inTissues and Plasma [J]. J Mol Diagn. 2009, 11(1):60-5.
