# Supplementary material for: Clinical value of ALU concentration and integrity index for the early diagnosis of ovarian cancer: A retrospective cohort trial
Source: PLoS One. 2018 Feb 5;13(2):e0191756. doi: 10.1371/journal.pone.0191756 (PMC5798773; doi:10.1371/journal.pone.0191756)
Supplement: S2 File — Trial study protocol-Chs.doc. (DOC) [file pone.0191756.s002.doc]

外周血循环DNA作为生物标志物在卵巢癌临床中的应用研究试验方案

试验目的：外周血循环游离DNA作为生物标志物在卵巢癌临床应用的价值

试验类别：诊断试验

申办单位：苏州大学

研究单位：苏州大学附属第二医院

试验负责人：李凯

主要研究者：张荣

试验日期：2012年11至2017年11月

联系人：张荣

电话：0512-67784769

**一、临床试验背景**

卵巢癌是妇科常见的恶性肿瘤之一，卵巢癌的死亡率居妇科肿瘤首位。70% 的患者发现时已是晚期( Ⅲ期和Ⅳ期)，虽经积极治疗，5 年生存率也仅为15%~20%，若早期(Ⅰ期) 发现，5 年生存率可达77% ~87%，若为分化良好的Ⅰ期患者，5 年生存率可达到94%[1]。因此，提高生存率的关键是早期诊断。特异而敏感的肿瘤标记物在辅助诊断卵巢癌中起重要作用。

近年来检测肿瘤患者外周血中游离DNA 的改变为肿瘤的早期诊断和复发监控提供了一种新的手段。游离DNA可存在于血浆或血清、脑脊液及滑膜液等体液中，是指一种游离于细胞之外的DNA，其中血液中的相应成分又称循环游离DNA或循环DNA。早在1947年Mandel等[2]就发现了人的血液循环中存在游离DNA。自1977 年leon等[3]发现肿瘤患者的血浆DNA 含量明显高于正常人后，关于肿瘤患者血中游离DNA的研究逐渐成为人们关注的热点。**对游离DNA的定性研究显示, 肿瘤患者的血浆DNA 具有与肿瘤细胞DNA一致的基因异常**[4-7]。

通过研究发现，原癌基因的突变，抑癌基因甲基化改变和微卫星不稳定性改变[8-9]，可以预测肿瘤患者的预后[10]。目前国外对血中游离DNA的研究多集中在肺癌、直肠癌、乳腺癌、前列腺癌、黑色素瘤等西方国家的“多发病”上，对卵巢癌的研究不多。Hagiwara等[11]对肺癌吸烟与不吸烟患者及健康人的游离DNA 分别进行了p53基因编码子248和249的突变检测，认为吸烟者游离DNA中p53基因突变可作为癌症的预测指标。Ryan等[12]对大量结肠癌患者的游离DNA研究表明，游离DNA中k-ras 基因突变的患者肿瘤复发率可达62. 5%。欧洲癌症和营养学前瞻性队列研究收集了准健康者至其癌症发生后的游离DNA，进行了k-ras基因12位编码子和p53基因的突变检测，这一系列研究包括膀胱癌、肺癌、上消化道癌等，**研究发现在肿瘤得到临床诊断前18. 3个月，在血中游离DNA中就可检测到相应的基因突变**，这种可预测性与肿瘤原发部位、类型和分期相关。

Frattini等[13]表明，游离DNA 定量、定性及定点的联合检测将提高肿瘤的临床诊断率。该研究观察了大肠癌患者手术前后的游离DNA 水平和游离DNA中k-ras和p16 的变化，发现术后游离DNA 水平逐渐下降，当肿瘤复发时快速升高，并重新可以检测到突变的k-ras和p16基因的甲基化。Melnikov[14]等用甲基化特异性PCR检测了卵巢癌患者手术的肿瘤组织和血浆中的甲基化差异，发现将BRCA1、HIC1、PAX5、PGR、THBS1基因合并检测，诊断卵巢癌的敏感性为85%，特异性为61%，其效率与直接应用肿瘤组织相当。Dobrzycka[15]等用PCR-RFLP的方法评价了血浆游离DNA与卵巢癌进展的关系，发现游离DNA的增加与肿瘤生存率降低有关。根据以上研究结果，我们希望可以通过类似的研究，发现卵巢癌患者游离DNA水平和相关基因的改变，获取相关中国人群血中游离DNA与卵巢癌病理变化的关系，为卵巢癌防治提供参考，并通过量化相关指标，提供可供复发监控的基因诊断方法。

目前研究充分表明了游离DNA 的变化与卵巢癌的关系密切。游离DNA 的发现及研究为疾病的非创伤性诊断、监测提供了可能，解决了肿瘤基因检测只能用肿瘤组织的标本限制，为肿瘤的诊断、治疗监控、预后随访等研究提供了简便途径。但是，由于游离DNA的微量存在，使得目前DNA 的提取比较困难，提取试剂昂贵。游离DNA 的检测方法不一，各自敏感度差异较大，游离DNA 检测的特异性低。因此，为方便游离DNA 的临床使用，需要建立实用性较强的检测方法。

本试验拟利用高保真DNA聚合酶的作用下巢式PCR和甲基化特异性的荧光定量PCR 检测游离血中游离DNA浓度，比较卵巢癌患者、卵巢良性囊肿患者、健康女性中血浆游离DNA 浓度的差别，并检测血中和卵巢癌手术标本中热点基因甲基化状态，以期发现外周血DNA的与卵巢癌组织的相关性。展望未来，卵巢癌游离DNA 的基础及临床研究必将成为受人瞩目的研究热点。

**二、试验目的**

评价血浆游离DNA作为生物标志物在卵巢癌诊断及预后方面的意义。

**三、试验设计**

本试验为回顾性队列研究。

**四、受试者的选择和退出**

**1.**入组标准：

年龄：20-80岁。

性别：女。

1）卵巢癌组：

具有病理学诊断依据的新发卵巢癌病例。

签署知情同意书。

2）良性卵巢囊肿组：

手术病理确诊的良性卵巢囊肿的病例。

签署知情同意书。

**2.**排除标准：

1）合并其他恶性肿瘤患者。

2）妊娠。

3）合并自身免疫性疾病。

**3.** 受试者中途撤出标准。

1）研究者从医学角度考虑受试者有必要中止试验。

2）患者自己要求停止试验。

**五、病例数及分组方法**

分为卵巢癌组、良性卵巢囊肿组及健康对照组。运用队列研究，卵巢癌组样本量50例，良性卵巢囊肿组50例，健康对照组50例。

**六、临床试验步骤：**

**（一）收集标本**

1. 卵巢癌组

1. 患者术前采集肘静脉血6ml，用EDTA抗凝。
2. 术中取新鲜卵巢癌组织标本1*1*1cm^3^，置-80℃冰箱中保存。
3. 卵巢癌患者于术后1周采血查血游离DNA浓度及完整性。之后按卵巢癌的随访标准，术后第一年每个月随访一次，术后第二年每三月随访一次，术后第3年每半年随访一次，术后第4、5年每年随访一次。每次随访时查血游离DNA浓度及完整性、血常规、生化常规，血CA-125，视临床情况行影像学或妇科彩超检查。共随访5年。

2. 良性卵巢囊肿组：

1) 患者术前采集肘静脉血6ml，用EDTA抗凝。

2) 术中取新鲜卵巢良性囊肿组织标本1*1*1cm^3^，置-80℃冰箱中保存。

3) 良性卵巢囊肿的病人于术后一月和术后半年各随访一次，以后每年随访一次，共随访5年。每次随访时测血浆游离DNA浓度及完整性、血CA-125，视临床情况行影像学或妇科彩超检查。

3. 健康对照组：

采集肘静脉血6ml，用EDTA抗凝，测血浆游离DNA浓度及完整性。每年随访一次，共随访5年。

**（二）血浆游离DNA浓度及完整性的检测**

1. 标本采集后2 h内送实验室。
2. 常温下3000r/min离心10 min，小心吸取上清血浆转移至3 ml离心管中，3000r/min离心10 min，吸取上清，置-80℃冰箱中保存。
3. 解冻血浆，血浆DNA抽提采用QIA amp Blood Mini Kit(Qiagen公司)，按说明书进行。
4. 游离DNA浓度及完整性测定：根据文献中给出的ALU基因的序列设计两对引物，一对引物为文献中已经给出的引物，扩增115bp长度，另一对引物自己设计，扩增长度为219bp，均由由上海生工合成。115bp:ALU 115-1：CCTGAGGTCAGGAGTTCGAG，ALU 115-2： CCCGAGTAGCTGGGATTACA。219bp: ALU 219-1：CACGCCTGTAATCCCAGCACTTT， ALU 219-2：ATCTCGGCTCACTGCAACCTCC。以提取的血浆DNA为模板，进行PCR扩增。以试剂盒内已知的标准品浓度可计算出实验标本的游离DNA浓度。计算血浆游离DNA完整性：血浆游离DNA完整性=ALU219-qPCR/（ALU115-qPCR+ ALU219-qPCR）。
5. 检测血中和卵巢癌手术标本中热点基因甲基化状态：利用高保真DNA聚合酶的作用下巢式PCR的方法，根据肿瘤相关基因（根据相关文献和NCBI中的序列信息）设计引物，做巢式PCR（高保真酶），将得到的PCR产物测序。根据测序结果再设计引物，进行甲基化特异性荧光定量PCR，提取健康人的、卵巢良性囊肿患者、卵巢癌患者手术前后的血浆游离DNA做模板，两端设计引物做荧光定量PCR，看不同组之间的游离DNA甲基化的数量是否有显著性差异。
6. 随访观察：三组实验对象于随访期内定期抽血检测血浆游离DNA浓度及完整性。

**（三）观察指标**

各组血浆游离DNA浓度及完整性、卵巢癌相关基因甲基化状态。

**七、统计分析**

本试验统计分析选用符合方案数据集，即所有符合试验方案要求的受试者数据进行统计分析。采用Dunnett’s 多组对比、t检验、Spearman’s相关性（一致性）检验、 ROC曲线分析和多分类 logistic回归双侧检验。

**八、试验段质量控制和保证**

本研究过程中，将由申请者指派的临床监查员定期对研究医院进行现场监查访问，以保证研究方案的所有内容都得到严格遵守和填写研究资料的正确。参加研究人员必须经过统一培训，统一记录方式与判断标准。整个临床试验过程均应在严格操作下进行。研究者应按病例报告表填写要求，如实、详细、认真记录CRF中各项内容，以确保病例报告表内容完整真实、可靠。临床试验中所有观察结果和发现都应加以核实，以保证数据的可靠性，确保临床试验中各项结论来源于原始数据。在临床试验和数据处理阶段均有相应的数据管理措施。

**九、伦理学要求**

本临床试验必须遵循赫尔辛基宣言和中国有关临床试验研究规范、法规进行。在试验开始之前，须经苏州大学附属第二医院伦理委员会批准认定该试验方案后方可实施。 每一位患者入选本研究前，研究医师有责任以书面文字形式，向其或其指定代表完整、全面地介绍本研究的目的、程序和可能的风险。应让患者知道他们有权随时退出本研究。入选前必须给每位患者一份书面患者知情同意书（以附录形式包括于方案中）。研究医师有责任在每位患者进入研究之前获得知情同意书，并以研究档案保留其中。

**资料保存：**使用后销毁

**参考文献：**

[1] Vergot e I, De Brabant e J, Fyles A, et al. Prognostic importance of degree of differentiation and cyst rupture in stage Ⅰ invasive epithelial ovarian carcinoma [J]. Lancet, 2001, 357(9251): 176-82.

[2] Mandel P, Met ais P. Les acides nucleiques du plasma sanguinchez I, home [J]. C R Acad Sci Paris, 1948, 142: 241-43.

[3] Leon SA, Shapiro B, Sklaroff DM, et al. Free DNA in the serum of cancer patient s and the effect of therapy [J]. Cancer Res, 1977, 37: 646- 50.

[4] Mirza S, S harma G, Prasad CP, et al. Promoter hypermethylation of TMS1, BRCA1, Eralpha and PRB inserum and tumor DNA of invasive ductal breast carcinoma patients [J] . Life Sci, 2007, 81(4): 280-7.

[5] Weaver KD, Grossman SA, Herman JG. Methylated tumor specific DNA as a plasma biomarker in patients with glioma [J]. Cancer Invest, 2006, 24(1): 35-40.

[6] Yang H J, Liu V W, Wang Y, et al. Detection of hypermethylated genes in tumor and plasma of cervical cancer patients [J] .Gynecol Oncol,2004, 93(2): 435-40.

[7]Sanchez-Cespedes M, EstellerM, Wu L, et al. Gene promoter hypermethylation in tumors and serum of head and neck cancer patients[J]. Cancer Res, 2000, 60(4): 892-95.

[8] Anker P, Stroun M. Circulating DNA in plasma or serum [J]. Medicina( B A ires), 2000, 60( 5Pt2) : 699-702.

[9] Shapiro B, Chak rabarty M, Cohn EM, et al. Determination of circulating DNA levels in patients with benign or malignant gastro intestinal disease[J] . Cancer, 1983, 51(11): 2116-20.

[10] Sozzi G, Conte D, Mariani L, et al. Analysis of circulating tumor DNA in plasma at diagnosis and during follow-up of lung cancer patients[J] . Cancer Res, 2001, 61(12): 4675-8.

[11] Hagiwara N, Mechanic LE, Trivers GE, et al. Quantitative detection of p53mutat ions in plasma DNA from tobacco smokers[J] . Cancer Res, 2006, 66(16): 8309-17.

[12] Ryan BM, Lefort F, M cmanus R, et al. A prospective study of circulating mutant KRAS2 in the serum of patients with colorectal neoplasia strong prognostic indicator in postoperative follow up [J] .Gut, 2003, 52(1): 101-8.

[13] Frattini M, Gallino G, Signoroni S, et al. Quantitative and qualitative characterization of plasma DNA identifies primary and recurrent colorectal cancer [J]. Cancer Lett, 2008, 263(2): 170-81.

[14]B. Dobrzycka, S. J. Terlikowski, M. Kinalski, et al. Circulating free DNA and p53 antibodies in plasma of patients with ovarian epithelial cancers[J]Ann. Oncol. 2011,22: 1133-40.

[15]A Melnikov, D Scholtens, AGodwin, et al. Differential Methylation Profile of Ovarian Cancer inTissues and Plasma [J]. J Mol Diagn. 2009, 11(1):60-5.
