# Supplementary figures and images for: Clinical value of ALU concentration and integrity index for the early diagnosis of ovarian cancer: A retrospective cohort trial
Source: PLoS One. 2018 Feb 5;13(2):e0191756. doi: 10.1371/journal.pone.0191756 (PMC5798773; doi:10.1371/journal.pone.0191756)

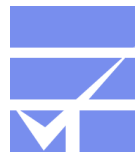

# CONSORT

TRANSPARENT REPORTING of TRIALS

## CONSORT 2010 Flow Diagram

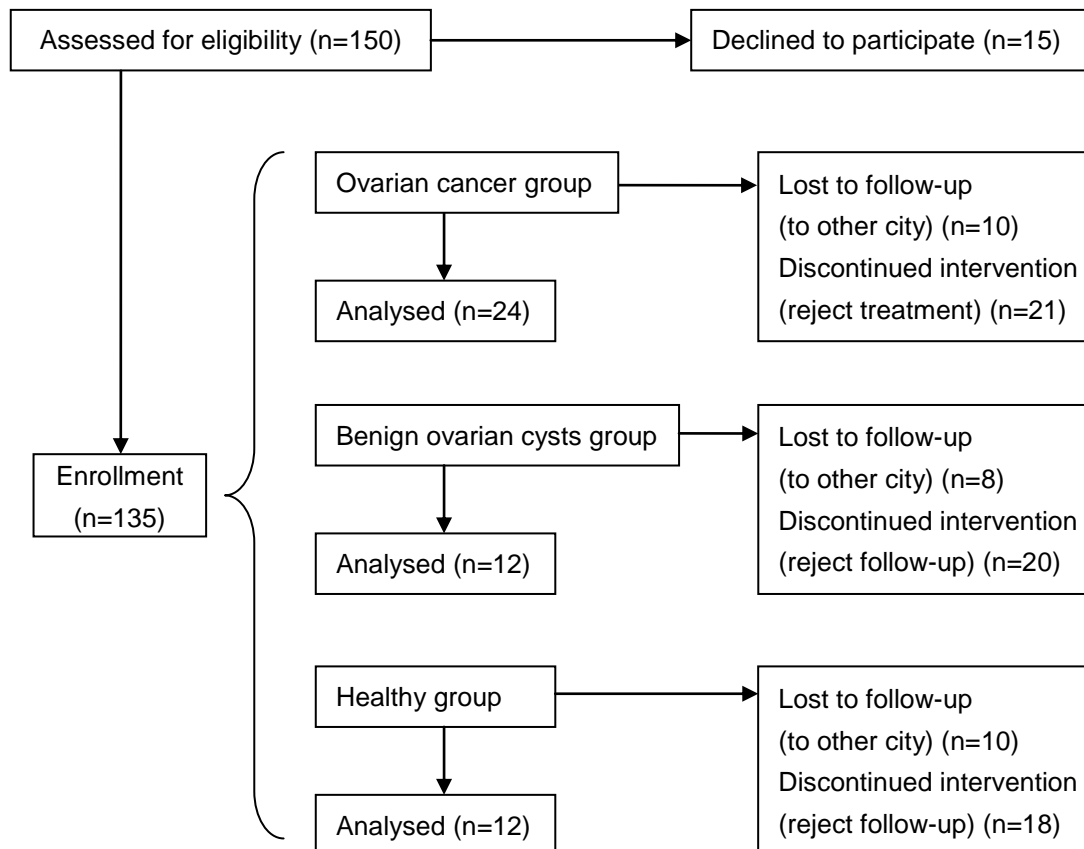

Supplement: S3 File — Flow chart.pdf. (PDF) [file pone.0191756.s003.pdf]
