# Supplementary material for: Clinical value of ALU concentration and integrity index for the early diagnosis of ovarian cancer: A retrospective cohort trial
Source: PLoS One. 2018 Feb 5;13(2):e0191756. doi: 10.1371/journal.pone.0191756 (PMC5798773; doi:10.1371/journal.pone.0191756)
Supplement: S4 File — Financial supports.pdf. (PDF) [file pone.0191756.s004.pdf]

INDEX

1. The National Natural Science Foundation of China-81301267 - Dr. Li Xiao

2. Suzhou Science and Technology Development Program - SZS201618 - Prof. Chungen Xing

3. The research foundation of the Second Affiliated Hospital of Soochow University - SDFEYQN201302 - Dr. Rong Zhang

1. The National Natural Science Foundation of China-81301267-Dr. Li Xiao

关于国家自然科学基金资助项目批准及有关事项的通知

苏州大学 肖莉先生/女士:

根据《国家自然科学基金条例》的规定和专家评审意见，国家自然科学基金委员会（以下简称自然科学基金委）决定批准资助您的申请项目。项目批准号：81301267，项目名称 反因子核酸酶靶向编辑 HER-2 基因的分子影像学评价，资助金额 23.00 万元，项目起止年月：2014 年 01 月至 2016 年 12 月，有关项目的评审意见及修改意见附后。

请尽早登录科学基金网络信息系统（https://isis.nsfc.gov.cn），获取《国家自然科学基金资助项目研究计划书》（以下简称计划书）并按要求填写。计划书电子文件通过科学基金网络信息系统（https://isis.nsfc.gov.cn）上传，由依托单位确认后，自然科学基金委进行审核；计划书纸质文件（一式两份）由依托单位审核并加盖单位公章后报送至自然科学基金委项目材料接收工作组。

自然科学基金委接收依托单位提交计划书电子版截止时间为 **2013 年 9 月 11 日 16 点前**，提交计划书电子修改版截止时间为 **2013 年 9 月 18 日 16 点前**；计划书纸质版于计划书电子版通过自然科学基金委审核后再行打印（建议双面打印），自然科学基金委接收计划书纸质版截止时间为 **2013 年 9 月 27 日 16 点前**。

请按照依托单位规定时间，及时将计划书电子版和纸质版先后提交依托单位进行确认审核。对于有修改意见的项目，请按修改意见及时调整计划书相关内容；如对修改意见有异议，须在计划书电子版报送截止日期前提出。计划书电子文件和纸质文件内容应当保证一致。

未说明理由且逾期不报计划书者，视为自动放弃接受资助。

附件：项目评审意见及修改意见

国家自然科学基金委员会

医学科学部

2013 年 08 月 15 日

项目批准号	81301267
申请代码	H1808
归口管理部门	
依托单位代码	21502108A0927-1735

813012671004704

国家自然科学基金委员会

资助项目计划书

资助类别：青年科学基金项目

亚类说明：

附注说明：

项目名称：反因子核酸酶靶向编辑HER-2基因的分子影像学评价

资助经费：23万元　　执行年限：2014.01-2016.12

负 责 人：肖莉

通讯地址：苏州市三香路1055号

邮政编码：　　电　　话：0512-67783692

电子邮件：lixiao0626@sina.com

依托单位：苏州大学

联 系 人：刘开强　　电　　话：0512-67507192

填表日期：2013年08月28日

国家自然科学基金委员会制

Version：1.004.704

国家自然科学基金委员会资助项目计划书填报说明

一、项目负责人收到《关于国家自然科学基金资助项目批准及有关事项的通知》（以下简称《批准通知》）后，请认真阅读本填报说明和自然科学基金相关项目及财务管理办法（查阅http://www.nsfc.gov.cn/），按《批准通知》的要求认真填写《国家自然科学基金委员会资助项目计划书》（以下简称《计划书》）。

二、填写《计划书》时要求科学严谨、实事求是、表述清晰、准确。《计划书》经主管科学部审核批准后，将作为项目研究计划执行和检查、验收的依据。

三、《计划书》简表部分自动生成，其他部分按以下要求填写：

（一）各类获资助项目都必须填写中、英文摘要及主题词，按批准经费填报经费预算表。

（二）正文撰写：

1. 对于面上项目、青年科学基金项目、地区科学基金项目，如果《批准通知》中没有修改要求的，只需选择“研究内容和研究目标按照申请书执行”即可；如果《批准通知》中明确要求调整研究内容的，须选择“根据研究方案修改意见更改”并填报相关修改内容。

2. 对于重点项目、重大项目、科学仪器基础研究专款项目及国家重大科研仪器设备研制专项（自由申请）项目，须选择“根据研究方案修改意见更改”，根据《批准通知》的要求填报研究内容，不得自行降低、更改研究目标（或仪器研制指标）或缩减关键的研究内容。此外，还要突出以下几点：
1)研究的难点和在实施过程中可能碰到的问题（或仪器研制风险），拟采用的研究方案和技术路线；
2)项目主要参与者分工，并请说明课题及合作单位之间的关系与分工。

3. 对于国家杰出青年科学基金、优秀青年科学基金和海外及港澳学者合作研究基金项目，须选择“根据研究方案修改意见更改”，按下列提纲撰写：
1）研究方向；
2）
结合国内外研究现状，说明研究工作的学术思想和科学意义（限两个页面）；
3）研究内容、研究方案及预期目标（限两个页面）；
4）分年度进度安排；
5）研究队伍的组成情况。

4. 对于其他类型项目，参照面上项目填报。

简表

<table>
<tr><td rowspan="6">申请者信息</td><td>姓 名</td><td>肖莉</td><td>性 别</td><td>女</td><td>出生年月</td><td>1981年01月</td><td>民 族</td><td>汉族</td></tr>
<tr><td>学 位</td><td colspan="3">硕士</td><td>职称</td><td colspan="3">助理研究员</td></tr>
<tr><td>电 话</td><td colspan="2">0512-67783692</td><td colspan="2">电子邮件</td><td colspan="3">lixiao0626@sina.com</td></tr>
<tr><td>传 真</td><td colspan="2"></td><td colspan="2">个人网页</td><td colspan="3"></td></tr>
<tr><td>工 作 单 位</td><td colspan="7">苏州大学</td></tr>
<tr><td>所 在 院 系 所</td><td colspan="7">附属第二医院</td></tr>
<tr><td rowspan="3">依托单位信息</td><td>名 称</td><td colspan="5">苏州大学</td><td>代码</td><td>21500601</td></tr>
<tr><td>联 系 人</td><td colspan="2">刘开强</td><td colspan="2">电子邮件</td><td colspan="3">kliu@suda.edu.cn</td></tr>
<tr><td>电 话</td><td colspan="2">0512-67507192</td><td colspan="2">网站地址</td><td colspan="3">http://www.suda.edu.cn/</td></tr>
<tr><td rowspan="3">合作单位信息</td><td colspan="7">单 位 名 称</td><td>代 码</td></tr>
<tr><td colspan="7"></td><td></td></tr>
<tr><td colspan="7"></td><td></td></tr>
<tr><td rowspan="7">项目基本信息</td><td>项 目 名 称</td><td colspan="7">反因子核酸酶靶向编辑HER-2基因的分子影像学评价</td></tr>
<tr><td>资 助 类 别</td><td colspan="4">青年科学基金项目</td><td>亚 类 说 明</td><td colspan="2"></td></tr>
<tr><td>附 注 说 明</td><td colspan="7"></td></tr>
<tr><td>申 请 代 码</td><td colspan="4">H1808</td><td colspan="3"></td></tr>
<tr><td>基 地 类 别</td><td colspan="7"></td></tr>
<tr><td>执 行 年 限</td><td colspan="7">2014.01-2016.12</td></tr>
<tr><td>资 助 经 费</td><td colspan="7">23.0000万元</td></tr>
</table>

项目摘要

中文摘要(500字以内):
乳腺癌是女性常见的恶性肿瘤，原癌基因HER-2的改变与乳腺癌发生发展密切相关。目前靶向肿瘤基因治疗及其在体多模态成像监测是分子影像学的研究热点。本项目拟采用已有前期工作在艾滋病基因治疗基础研究中使用的新型分子反因子核酸酶，特异性靶向剪辑HER-2基因，研究阻断其信号通路达到靶向治疗乳腺癌的目的。实验采用荧光显微镜、动物活体成像仪、MRI多模态成像监测反因子核酸酶剪切效率和对肿瘤的治疗作用。研究内容包括：1.研发高效剪辑HER-2基因的反因子核酸酶，经腺病毒包装，行离体及在体癌细胞抑制和杀伤实验；2.用多模态成像监测反因子核酸酶对HER-2的剪辑效率和对癌细胞的抑制、杀伤能力，与荧光显微镜、流式细胞仪和病理学等检查相对照，评价多模态成像对该技术的监测效果和反因子核酸酶技术用于治疗乳腺癌的可行性。

关键词(不超过5个，用分号分开): 反因子核酸酶 ;多模态成像 ;乳腺癌 ;基因治疗 ;人表皮生长因子受体2

Abstract(limited to 500 words):
Breast Cancer is one of the most common female malignant tumors. Gene therapy as a novel and effective therapeutic approach, paved an avene to the the clincal treatments of breast cancer without the severe adverse events. As HER-2 signaling pathway plays a vital role in the development of breast cancer. Transcription Activator-Like Effector Nucleases (TALENs) is used to konck-out the HER-2 gene in this study. This grant application include:1.preparing and selecting the best HER-2 element, cloning and seleting the most efficient TALENs targeting HER-2 gene and preparing its adenoviral form; 2. testing the effects in in vitro culture cancer cells and evaluating the in vivo effects on breast cancer of TALENs using florescent scanning, MRI, optical imaging in multi-modal imaging systems.

Keywords(limited to 5 keywords,seperated by;): TALENS ;multi-modal imaging ;breast cancer ;gene therapy ;HER-2

项目组主要成员

编号	姓名	出生年月	性别	职称	学位	单位名称	电话	电子邮件	项目分工	每年工作时间（月）
1	肖莉	1981.01	女	助理研究员	硕士	苏州大学	0512-67783692	lixiao0626@sina.com	项目负责人	7
2	周丽娟	1971.1	女	副主任医师	硕士	苏州大学	0512-67783692	zlj1971sz@126.com	多模态成像评价	6
3	张荣	1980.5	女	主治医师	硕士	苏州大学	0512-67783692	rongwhu@163.com	评估靶基因表达谱	6
4	潘韵芝	1988.1	女	硕士生	学士	苏州大学	0512-67783692	yunzhipan@126.com	TALENS表达评估	8
5	孙爱娟	1989.5	女	硕士生	学士	苏州大学	0512-67783692	sunaijuan2011@163.com	剪辑效率离体评估	8
6	徐惠芬	1989.8	女	硕士生	学士	苏州大学	0512-67783692	huifenxu@163.com	裸鼠荷瘤实验	8
7	刘彬	1989.2	男	硕士生	学士	苏州大学	0512-67783692	liubin198866@163.com	剪辑效率在体评估	8

总人数	高级	中级	初级	博士后	博士生	硕士生
7	1	2	0	0	0	4

2. Suzhou Science and Technology Development

Program-SZS201618-Prof. Chungen Xing

苏州市科学技术局
苏 州 市 财 政 局

苏科资〔2016〕135 号

苏财教字〔2016〕59 号

关于下达苏州市 2016 年度第六批科技发展计划（科技设施、科技创新战略研究基地）项目及经费的通知

张家港市、常熟市、昆山市、吴江区、相城区、姑苏区、工业园区、高新区科技局、财政局，各有关单位:

现将苏州市 2016 年度第六批科技发展计划（科技设施、科技创新战略研究基地）项目经费下达给你们，相应增列张家港市、常熟市、昆山市、吴江区、相城区、姑苏区、工业园区、高新区“应用技术研究与开发”（政府收支分类科目编码: 2060402）预算支出指标。本批下达项目共计 23 项，共下达科技创新专项资金 1140 万元，本年度拨款 800 万元。

请各主管部门收到通知后，通知项目承担单位登录“苏州科

技计划项目管理系统”，网上填写《苏州市科技计划项目合同》，一式四份打印，与主管部门、市科技局签订纸质合同，并办理相应手续。请严格按照市科技计划管理办法和科技经费管理的有关规定，加强项目的组织协调和实施管理，确保科技经费专款专用，促进项目按期完成，并及时将有关项目执行情况报市科技局、财政局。

请项目承担单位按财政支出绩效评价要求，定期将进展情况报苏州市科技局、财政局，做好科技统计和数据上报工作。

附件：1. 苏州市 2016 年度第六批科技发展计划（科技设施、科技创新战略研究基地）项目经费分配表

2. 苏州市 2016 年度第六批科技发展计划（科技设施、科技创新战略研究基地）项目表

苏州市科学技术局　　　　苏州市财政局

二〇一六年六月三十日

主题词：项目　拨款　通知

抄　送：中国人民银行苏州分行国库处

序号	项目编号	项目名称	项目简介	承担单位	项目负责人	主管部门	起止时间	总经费	市拨款	本年度拨款
			军人才及 15 名中青年学术骨干，形成 30 人的研发团队，其中硕士以上人员占比 80%，高级以上职称 10 人。							
7	SZS201615	苏州市儿童白血病重点实验室	开展儿童急、慢性白血病/淋巴瘤的早期预警、早期诊断的研究以及新型抗体靶向药物的筛选和研发。项目期内，实验室面积达 4000 平方米，在国际著名杂志或会议上发表高质量的研究论文 3 篇以上，在国内一流杂志发表论文 5 篇以上，申报和开展国家级、省级、市级医学继续教育项目各 1 项以上，科学研究项目 3 项以上；申报专利 2 项以上；培养研究生 10 人以上，申报增列硕士生导师 2 名以上，增列博士生导师 1 人以上。	苏州大学附属儿童医院	胡绍燕	市卫计委	2016.07.01-2019.06.30	600	30	30
8	SZS201616	苏州市肿瘤微环境病理学重点实验室	开展消化和呼吸系统肿瘤研究。项目期内，改建 2500 平方米的实验室，建设肿瘤样本库管理平台、组织与分子病理学技术平台、分子生物学技术平台；形成 50 人的研发团队，发表 SCI 论文 15 篇以上，申请专利 10 项以上；申请获得国家自然科学基金 6 项以上，申办国际和国内精准医疗相关学术会议各 1 次。	苏州大学	时玉舫	苏州大学	2016.07.01-2019.06.30	3000	30	30
9	SZS201617	苏州市呼吸疾病重点实验室	开展呼吸疾病的深入研究。项目期内，新增实验室面积 500 平方米，新增流式细胞仪 1 台，发表 SCI 论文 10 篇以上，国家级期刊学术论文 5 篇以上，获市、厅级科技成果奖 2 项以上、省部级奖项 1 项；在高层次（国外）学术会议上作专题报告 3 次以上；培养博士研究生 3 名以上，硕士研究生 10 名以上；新增省级以上课题 2 项以上，参加国内专家共识撰写 1 部以上；举办国家级学术会议 1 次以上，国家级继续教育学习班 3 次以上；申报专利 1 项以上。	苏州大学附属第一医院	黄建安	市卫计委	2016.07.01-2019.06.30	1400	30	30
10	SZS201618	苏州市精准肿瘤医学重点实验室	开展针对精准肿瘤医学中的大样本人群与特定疾病类型的靶向位点分析与鉴定、验证与应用等领域的研究。项目期内，实验室面积达 1100 平方米，完成多中心基因数据库平台、肿瘤基因检测技术研发平台、肿瘤细化基因分型临床转化平台、靶向药物分子监测平台的建设，完成基于蓝白斑的突变筛选技术、体外基因转换开关技术、串联 PCR、限制性内切酶及测序技术；发表 SCI 论文 2 篇以上，影响因子累积 6 分以上，申请国家专利 2 项以上，联合培养博士生不少于 3 名，培养中高级以上职称技术专家不少于 5 名。	苏州大学附属第二医院	邢春根	市卫计委	2016.07.01-2019.06.30	500	30	30

3. The research foundation of the Second Affiliated Hospital of Soochow University-SDFEYQN201302-Dr. Rong Zhang

苏州大学附属第二医院文件

附二院〔2013〕60号

关于下达2013年苏州大学附属第二医院科研预研基金第二批项目和经费的通知

各科室:

根据《苏州大学附属第二医院科研预研基金管理办法(试行)》，经有关科室组织申报，院内外专家的网评初选及最终的答辩评审，共12个项目确立为我院第二批医院科研预研基金项目。

本次项目经费分两次下拨，首期拨款 50%。请有关科室及项目负责人认真组织安排，确保项目的顺利实施。

特此通知。

附件：2013 年医院科研预研基金第二批项目清单

苏州大学附属第二医院

2013 年 9 月 5 日

苏州大学附属第二医院办公室 2013年9月5日印发

附件

2013年医院科研预研基金第二批项目清单

单位：　万元

编号	科室	姓名	项目类别	批准经费	本次下达	项目名称	开始时间	结束时间
SDFEYQN1301	妇产科	刘利芬	青年职工预研基金项目	1	0.5	褪黑素对大鼠心肌缺血再灌注心肌细胞胀亡的影响及线粒体通透性转换孔在其中的作用	2013-8-1	2015-7-30
SDFEYQN1302	妇产科	张荣	青年职工预研基金项目	1	0.5	利用甲基化差异建立21-三体综合征无创产前诊断新方法	2013-8-1	2015-7-30
SDFEYQN1303	骨科	秦建忠	青年职工预研基金项目	1	0.5	旋髂深动脉髂骨组织瓣的应用解剖及三维数字化设计	2013-8-1	2015-7-30
SDFEYQN1304	呼吸科	杜紫燕	青年职工预研基金项目	1	0.5	TIGAR对p53介导的肺癌细胞A549死亡通路的调整及机制研究	2013-8-1	2015-7-30
SDFEYQN1305	内分泌科	张弘弘	青年职工预研基金项目	1	0.5	硫化氢介导糖尿病性胃部痛觉高敏的分子机制研	2013-8-1	2015-7-30
SDFEYQN1306	神经内科	胡华	青年职工预研基金项目	1	0.5	Notch信号通路在抑郁症小鼠脑血管新生中的作用机制研究	2013-8-1	2015-7-30
SDFEYQN1307	胸外科	桑永华	青年职工预研基金项目	1	0.5	体外循环通过影响铁稳态对心肌的损伤及其机制	2013-8-1	2015-7-30
SDFEYQN1308	整形美容外科	伍丽君	青年职工预研基金项目	1	0.5	压力差改善狭长窄蒂皮瓣血供的实验研究	2013-8-1	2015-7-30
SDFEYQN1309	病理科	董赟	青年职工预研基金项目	1	0.5	Slug在结肠癌血管生成拟态形成中的作用	2013-8-1	2015-7-30
SDFEYQN1310	眼科	金吉	青年职工预研基金项目	1	0.5	下丘脑弓状核对眼压的影响及其与阿片肽关系的研究	2013-8-1	2015-7-30
SDFEYBS1301	内分泌科	马志敏	博士、留学归国人员预研项目	3	1.5	肌细胞因子Irisin调节肝脏甘油三酯代谢的作用和机制研究-2013-0625-医院	2013-8-1	2015-7-30
SDFEYBS1302	肾内科	孔淑敏	博士、留学归国人员预研项目	3	1.5	肾结石形成中CaSR对大鼠肾脏髓袢升支粗段管周膜氯通道的调控	2013-8-1	2015-7-30
